# O12.8. LOWER FAMILY INCOME PREDICTS PSYCHOTIC EXPERIENCES IN A COMMUNITY SAMPLE OF YOUTHS IN BRAZIL: RESULTS FROM A 3-YEAR FOLLOW-UP STUDY

**DOI:** 10.1093/schbul/sby015.276

**Published:** 2018-04-01

**Authors:** Lais Fonseca, Luisa von Nielander, Felipe Argolo, Pedro Pan, Rodrigo Bressan, Ary Gadelha

**Affiliations:** 1 Universidade Federal de Sao Paulo; 2 Federal University of Sao Paulo, University of Sao Paulo Medical School; 3 Laboratório Interdisciplinar de Neurociências Clínicas (LiNC), Universidade Federal de São Paulo

## Abstract

**Background:**

Up to 17% of community youths, from 9 to 12 years old, report subthreshold psychotic experiences (PE). Besides increasing conversion risk to psychotic disorders, PE also predicted suicide attempts and were associated with more severe general psychopathology. Understanding predisposing factors to PE during development can inform on risk to mental disorders, a crucial step to future prevention interventions. Adversity in early life has been associated with psychotic symptoms, and low socioeconomic status (SES) is an established environmental risk factor for several mental disorders. However, few studies investigated the effect of low SES on PE risk in youths, prospectively. This topic is highly relevant in underprivileged countries like Brazil, where large populations are exposed to poverty. We hypothesized that low income at baseline would predict later report of PEs.

**Methods:**

We analyzed data from the Brazilian High Risk Cohort Study for Psychiatric Disorders (HRC), in which 2,512 youths (6–12 years old, mean age at baseline 9.7 years, SD = 1.92; 53,1% male) completed the baseline assessment and 2,012, the 3-year follow-up (T1). PE were assessed at each time-point through two sources of information: parental report, using the Child Behavior Checklist (CBCL), and youth self-report, using the positive dimension of the Community Assessment of Psychic Experiences (CAPE). A single latent variable for each time-point was created to encompass both sources of information using Confirmatory Factor Analysis, yielding good model fit. Total family income (T0) was correlated to the psychotic latent variable (T1), controlling for age, gender and baseline report of PE. Then, we investigated how any mental disorder diagnosis and exposure to trauma, two possible confounding factors, affected the results. The Development and Well-Being Assessment (DAWBA) was used to investigate mental disorders diagnoses. No conversion to psychotic disorder was observed at the follow-up. A latent variable encompassing parental and youths reports, measured by Childhood Trauma Questionnaire (CTQ), was used as trauma exposure.

**Results:**

The mean family income reported was approximately US$ 394 per month (p25 = US$ 195, p50 = US$ 307, p75 = US$ 516). Income (baseline) and PE (T1) had a negative significant correlation (pcorr = -0.064; df = 2004; p = 0.004). Subjects with any mental disorder reported PE more frequently, either at baseline or at the follow-up. Low income inversely correlated with trauma, whereas trauma was strongly associated with PE and having any mental disorder at both time points. The correlation between income and PE did not remain significant, after controlling for any mental disorder (T1) (pcorr = -0.049; df = 1597; p = 0.050) or trauma exposure (T0) (pcorr = -0.043; df = 2003; p = 0.053).

**Discussion:**

Other studies have previously reported a positive correlation between adversity and PE, but few have described a specific association between family income and psychotic experiences. Our additional analyses suggest that this is not specific, considering the influence of non-psychotic mental disorders, and mediated by low-income related exposures, i.e. childhood trauma. Even not surviving multivariate analyses, income could work as a proxy for adversity exposures and may be useful as a tool to define populations at risk for psychotic and non-psychotic mental disorders. We expect that the longer follow-up assessments help us answer the questions raised.

